# Using creativity and the arts to promote mental health in youth living with HIV in South Africa

**DOI:** 10.4102/sajhivmed.v25i1.1656

**Published:** 2024-12-20

**Authors:** Jacqueline Hoare, Rebecca Sher, Kathryn R. Cullen

**Affiliations:** 1 Department of Psychiatry, Faculty of Health Sciences, University of Cape Town, Cape Town, South Africa; 2 Department of Paediatrics, Faculty of Health Sciences, University of Cape Town, Cape Town, South Africa; 3 Department of Psychiatry and Behavioural Sciences, Medical School, University of Minnesota, Minneapolis, United States of America

**Keywords:** HIV, adolescent, youth, mental health, creative arts

## Abstract

Access to adolescent-friendly, culturally relevant and stigma-free mental health support is essential for reducing the long-term psychological, social and economic challenges of mental illness of youth living with HIV (YLWH). Now more than ever, innovative task-shifting interventions, through which non-mental health professionals provide mental health support to YLWH, need to be explored and supported. While many of these have considered shifting tasks to nurses, tapping into the wisdom and inspiration from artists in the community where YLWH are living could represent a novel and potentially powerful task-shifting strategy.

In this opinion piece, we propose that the arts could be explored in future studies as a promising avenue for mental health interventions for YLWH in South Africa. Better Together is a peer-support intervention for youth living with chronic illness, which has been published previously by our team.

As part of the discussion, we share feedback about the creative arts component of the Better Together groups provided by Better Together participants themselves.

Overall, this feedback yielded several key insights which further underscore the idea that providing youth with opportunities to engage in creative arts in a group setting may represent a promising platform for addressing mental health in YLWH in South Africa.

Specifically, we learned that (1) youth enjoyed the opportunity to engage creatively, (2) these experiences helped them connect with others, (3) they gained new insights and perspectives about themselves and their lives, and (4) they experienced a positive impact on their mood and well-being.

**What this study adds:** Providing YLWH with opportunities to engage in creative arts in a group setting may represent a promising platform for addressing mental health in YLWH in South Africa. We learned that YLWH gained new insights and perspectives about themselves and their lives; and experienced a positive impact on their mood and well-being.

## Introduction

Based on evidence that providing mental health care to those living with HIV can improve both mental health and HIV outcomes, including adherence to treatment,^1,2^ research investigating ways to integrate mental health care into the care of people living with HIV has become a public health priority in South Africa.^3^ Unsurprisingly, addressing mental health in youth living with HIV (YLWH) 15–24 years in South Africa is no simple task, and one which faces many barriers. First, these youth already have a burden of treatment for their HIV (e.g. youth already feel that they have to take ‘too many pills’ and may resist the idea of additional pills for mental health). Second, some communities in South Africa may hold a general distrust of interventions that are perceived as culturally insensitive,^4^ fueled by a long-standing history of racist policies and, historically, years of targeted government misinformation regarding the HIV epidemic. Third, psychologists and psychiatrists are in short supply, with 0.28 psychiatrists and 0.32 psychologists per 100 000 population working in the public health sector in South Africa.^5^ These alarming shortages will be worsened by the new austerity measures imposed by the South African National Government in the 2024/25 budget for the public health wage bill. New strategies are needed that can address these multiple barriers.

In this opinion piece we discuss the possibility of using arts-based interventions, which may represent a promising avenue for mental health treatment for YLWH in South Africa. A recent review highlighted the potential value of arts-based interventions to address the global youth mental health crisis, and recommended that future research:

(1) elevate and prioriti[*s*]e youth voice, (2) develop core outcome measures, (3) identify and analy[*s*]e successful models around the globe, and (4) generate clear funding pathways for research and translational efforts.^6^

They also noted that ‘Worldwide implementation of arts- and culture-based strategies to address youth mental health will provide critical resources to support the health, wellbeing and flourishing of countless youth across the globe’.^6^ In particular, music and dance are a central part of life in many cultural groups across South Africa,^7^ and visual arts have been practised in South Africa since ancient times.^8^ For centuries, art and music have played a significant role in cultural expression, communication, and storytelling in the region.^9^ Therefore, inviting YLWH to engage in arts and music could be a culturally sensitive entry point for mental health intervention and HIV care engagement in South Africa, particularly if youth are invited to co-create the intervention along with local artists and arts therapists.

One illustrative example is the Lalela Project, running in South Africa since 2010 at several sites in the Western Cape and Gauteng, which uses art therapy to help children and adolescents affected by poverty and violence to express their emotions and build connections with their peers. The Lalela Project recognises and emphasises the importance of creativity in a child’s life.^10^ Behavioural neuroscience research agrees with this assertion, and shows that intelligence and creative thinking rely on similar neural and cognitive systems.^11,12^ The deep connections between intelligence and creativity emphasise the similarities between solving problems and thinking flexibly, critically, and playfully.^13^ The creative arts can also play a vital role in fostering social connections and cultural identity within African communities, and provide a culturally relevant and accessible approach to addressing mental health concerns.

Emerging evidence supports how incorporating ways to support creative arts engagement in the care of YLWH has the potential to benefit their mental health and wellbeing.^6^ In a recent rapid review of mental health interventions for youth in Southern Africa, the authors note the potential advantages of interventions that indirectly address mental health for hard-to-reach groups to avoid some of the stigma connected to ‘mental health’ which can otherwise act as a barrier to participation.^6^ Four of the HIV-related interventions described in the review focused on therapy through storytelling, crafting, art, or film, where the participants could explore their memories in a safe environment, working through difficult emotions and negative themes of their situation. The review also noted that one of the main mechanisms for success revealed in a range of interventions was the inclusion of youth in the design and implementation of interventions.^14^

## Opinion piece objective

In this opinion piece, we propose that the arts could be explored in future studies as a promising avenue for mental health interventions for YLWH in South Africa. Better Together is a peer-support intervention for youth living with chronic illness, which has been published previously by our team.^15^ As part of the discussion in the opinion piece we share feedback about the creative arts component of the Better Together groups provided by Better Together participants themselves.

## Better Together adolescent programme at Groote Schuur Hospital

The Better Together programme provides weekly facilitated group sessions open to all youth, ages 13–24 years, who are living with a chronic condition and receiving care at Groote Schuur Hospital (GSH) in Cape Town, South Africa. Groote Schuur Hospital is a large public health academic hospital where care is provided free to patients through the South African governmental health services. Since 2017, Better Together has helped youth with chronic conditions, including HIV, to (1) build social networks that enhance support, (2) develop a sense of belonging with peers, (3) create a space where they can share their experience of living with and managing chronic illness, and (4) build empathy.^15^ Groups are facilitated by volunteer youth peer mentors who are also living with a chronic condition. The programme is popular and sustainable, as shown by our attendance statistics. Between August 2017 and November 2023, there were 2390 group visits involving 553 individuals, of which 58% were young women. The median age was 18 (interquartile range [IQR]: 16–21) years. Clinic attendees came from the following GSH clinical services: HIV (*n* = 222; 40%), Psychiatry (*n* = 116; 21%), and Renal (*n* = 103; 19%). The remainder came largely from the adolescent ward (diverse conditions), as well as endocrine, respiratory and gastrointestinal medicine clinics. We recently published the results of a mixed methods study to assess the acceptability, feasibility and preliminary mental health impacts of Better Together for youth living with chronic illnesses, including HIV.^15^ The previously published mixed-methods pilot study enrolled 58 young patients, ages 13–24 years, at GSH. In-depth interviews elicited perspectives of 20 young people in relation to their participation in the Better Together programme. Self-reported resilience, as well as attitudes towards illness, stigma, and mental health, were captured via established measures.^15^ Compared to young patients with chronic illness at GSH in Cape Town who did not participate in this programme, those who attended Better Together showed statistically significantly higher individual-level resilience, a more positive attitude towards their illness, lower internalised stigma, and a more positive self-concept.^15^ The probability of being screened positive for depression was lower for Better Together participants compared to controls; the probability of a positive anxiety screening was also significantly lower.^15^ All interviewees valued being able to compare treatment regimens and disease management with peers living with other conditions. Adolescents living with HIV found that understanding difficulties faced by those with other illnesses helped them accept their own, and lessened feelings of disconnection.^15^

## Better Together creative arts feedback from participants

The Better Together service includes linking YLWH to a peer mentor who is also living with HIV, as well as weekly facilitated group sessions. One session per month is facilitated by a Health Professions Council of South Africa-registered drama therapist, and another movement session per month is facilitated by Kids Kicking Cancer, a well-established non-profit organisation in South Africa which uses movement and mind-body techniques of Martial Arts instruction, breath work and meditation to empower children beyond the pain and discomfort of chronic disease.

The need for ‘youth friendly’ approaches, designed with input from local youth to care for YLWH, has been increasingly recognised.^16^ In the spirit of gaining insights from YLWH in the design of their care, and to further illustrate the potential value of utilising the creative arts in the care of YLWH, here we further report the feedback provided by the Better Together participants on the content of the programme. Participants are asked every 6 months to complete an anonymous feedback form which is used by the clinicians at GSH who run the service to modify the content of the programme according to the input gained from the youth themselves.

In response to our question, ‘What type of sessions have been your favourite and why?’, 18 out of 36 respondents mentioned drama therapy and movement sessions as being their favourite. Common themes in the answers to this question were that participation in these sessions allowed them to have fun while expressing themselves creatively, assisted with the development of self-awareness, feeling connected to others living with chronic illness, the value of being connected to others when feeling down, and being active. This theme is exemplified by the following statements provided anonymously by programme participants:

‘I enjoy when we created art to describe what we like, who we are, and what we want or need in life and our basic daily activities.’

‘I enjoyed the drama lesson; it was really fun and showed a lot of creativity amongst us.’

‘Everything. It was insightful, especially drama therapy, it was creative from most memories.’

‘Social and karate these groups have given me a chance to see that life when is sour you need to sociali[s]e with others.’

‘Drama I found very fun it allowed the group to be creative and think outside of serious life.’

We also asked the question, ‘Did the drama and/or movement sessions in Better Together help you? If you did find them beneficial, how did they help you?’ A common theme of the responses was that youth felt that their participation in drama and movement group sessions improved their mental wellbeing, and broadened their perspectives and points of view. The sessions improved their ability to express themselves, and improved emotional regulation and self-awareness. This theme is exemplified in the following statements from the anonymous feedback:

‘Yes it has helped me and I do find it beneficial, the drama it’s a different therapy something I wasn’t aware of and being introduced to it has helped, it is differ to the “normal” therapy and sometimes it gives a person a different perspective.’

‘To stay calm and take it to account that I’m not the only going through stuff, have hope even if there’s none to be, make the best out of situation.’

‘Yes the sessions have helped me, expressing myself creatively and it opened my mind to how people see different things looking at the same thing for example with one of the drama session we had to use images to tell people about ourselves, we all saw different meanings but at the same time there were things in common.’

‘A lot, every session reflects on who we are at the core and we need nature our true self, learning that the power to be better everyday is within us.’

‘Yes the drama sessions have helped me because during Karate you don’t think about your problems it helps you relax and calms you down.’

‘It helped my mental health to find ways to deal with stress and ways to calm myself down when I am nervous.’

‘I find the drama sessions very beneficial because they bring joy and excitement in the group. They helped me understand my thoughts and views about other things. Every drama sessions [*sic*] for me meant me being at ease and free and help me get out of my comfort zone.’

Overall, this feedback yields several key insights which further underscore the idea that providing youth with opportunities to engage in creative arts in a group setting may represent a promising platform for addressing YLWH in South Africa. Specifically, we learned that (1) youth enjoyed the opportunity to engage creatively, (2) these experiences helped them connect with others, (3) they gained new insights and perspectives about themselves and their lives, and (4) they experienced a positive impact on their mood and wellbeing.

## Conclusion

We present these results as evidence supporting the need for research to further explore the feasibility and efficacy of arts-based interventions to address the complex needs of YLWH in South Africa. Access to youth-friendly, culturally relevant, and stigma-free mental health support is essential in reducing the long-term psychological, social and economic challenges of mental illness particularly in those with co-morbid HIV infection. Now more than ever, innovative task-shifting interventions, through which non-mental health professionals provide mental health support to YLWH, need to be explored and supported. While many of these have considered shifting tasks to nurses, tapping into the wisdom and inspiration from artists in the community where YLWH are living could represent a novel and potentially powerful task-shifting strategy.
